# Long-Term Outcomes of Three-Dimensional High-Dose-Rate Brachytherapy for Locally Recurrent Early T-Stage Nasopharyngeal Carcinoma

**DOI:** 10.3389/fonc.2019.00278

**Published:** 2019-04-26

**Authors:** Yanzhu Lin, Yi Ouyang, Zhiyuan Lu, Yonghong Liu, Kai Chen, Xinping Cao

**Affiliations:** ^1^Guangdong Key Laboratory of Nasopharyngeal Carcinoma Diagnosis and Therapy, State Key Laboratory of Oncology in South China, Department of Radiation Oncology, Sun Yat-Sen University Cancer Center, Collaborative Innovation Center for Cancer Medicine, Guangzhou, China; ^2^Department of Oral and Maxillofacial Surgery, First Affiliated Hospital, Sun Yat-sen University, Guangzhou, China

**Keywords:** nasopharyngeal carcinoma, external beam radiotherapy, 3D brachytherapy, local recurrent, high-dose rate

## Abstract

**Background:** Brachytherapy (BT) is one of the techniques available for retreatment of patients with locally recurrent nasopharyng eal carcinoma (rNPC). In this study, we evaluated the treatment outcome and late toxicities of three-dimensional high-dose-rate brachytherapy (3D-HDR-BT) for patients with locally rNPC.

**Materials and Methods:** This is a retrospective study involving 36 patients with histologically confirmed rNPC from 2004 to 2011. Of the 36 patients, 17 underwent combined-modality treatment (CMT) consisting of external beam radiotherapy (EBRT) followed by 3D-HDR-BT, while the other 19 underwent 3D-HDR-BT alone. The median dose of EBRT for the CMT group was 60 (range, 50–66) Gy, with an additional median dose of BT of 16 (range, 9–20) Gy. The median dose for the 3D-HDR-BT group was 32 (range, 20–36) Gy. The measured treatment outcomes were the 5- and 10-year locoregional recurrence-free survival (LRFS), disease-free survival (DFS), overall survival (OS), and late toxicities.

**Results:** The median age at recurrence was 44.5 years. The median follow-up period was 70 (range, 6–142) months. The 5-year LRFS, DFS, and OS for the entire patient group were 75.4, 55.6, and 74.3%, respectively, while the 10-year LRFS, DFS, and OS for the entire patient group were 75.4, 44.2, and 53.7%, respectively. The 10-year LRFS in the CMT group was higher than that in the 3D-HDR-BT-alone group (93.8 vs. 58.8%, HR: 7.595, 95%CI: 1.233–61.826, *p* = 0.025). No grade 4 late radiotherapy-induced toxicities were observed.

**Conclusions:** 3D-HDR-BT achieves favorable clinical outcomes with mild late toxicity in patients with locally rNPC.

## Introduction

Nasopharyngeal carcinoma (NPC), a tumor of epithelial origin, is a malignant disease of the head and neck common in southern China, especially in Guangdong province (1). As a result of advances in modern imaging and irradiation techniques, the 5-year overall survival (OS) of patients with newly diagnosed NPC without metastasis has reached 75% after external beam radiotherapy (EBRT) in Asia (2, 3). However, local recurrence, which occurs in 18–40% of patients, remains a major reason for treatment failure (4, 5). Thus, treatment of patients with recurrent NPC (rNPC) is a major challenge for clinicians. Current therapies for locally rNPC include surgery, stereotactic radiosurgery, conventional radiotherapy, intensity-modulated radiation therapy (IMRT), and brachytherapy (BT) (6).

Patients with rNPC should usually be considered for reirradiation, which can be performed by intracavitary brachytherapy (ICBT) with or without EBRT. BT offers the possibility of high-dose irradiation with a rapid dose fall-off beyond the target volume, thereby sparing surrounding critical structures (7). Numerous studies have demonstrated the categorical benefit of BT in achieving favorable long-term local control and improving the survival outcome of patients with rNPC (8, 9). Computed tomography (CT)-guided high-dose-rate brachytherapy (HDR-BT) is an afterloading technique in which an ^192^Ir-source is temporarily inserted through catheters placed under CT guidance into the targeted tumor volume (10). CT-HDR-BT has the major advantage of delivering a high dose of radiation to cancers of the nasopharynx, and it probably reduces the amount of unnecessary damage caused to surrounding healthy tissues. However, the efficacy of 3D-HDR-BT in the treatment of rNPC remains unclear.

The present study is a retrospective analysis of 36 patients with locally rNPC, who were treated with 3D-HDR-BT alone or in combination with EBRT. Here, we report the long-term outcomes and treatment-related toxicities of the patients with locally rNPC.

## Materials and Methods

### Patients

This was a retrospective study conducted at the Department of Radiation Oncology, Sun Yat-sen University Cancer Center. For the period April 2004 to August 2011, 36 patients with locally rNPC without evidence of distant metastasis were eligible for the trial. The study protocol was approved by the Ethics Committee of Sun Yat-sen University Cancer Center and written informed consent were provided by patients. After histopathological verification of recurrence, chest radiography, abdominal ultrasonographic examination, bone scans, and CT or magnetic resonance imaging of the head and neck were performed. Patients with histologically or cytologically proven NPC, without distant metastases, had first recurrence and with Eastern Cooperative Oncology Group (ECOG) performance status of less than grade 2 were included. Local recurrence was defined as the presence of a tumor growth for 3 months or longer after complete regression, after completion of primary treatment.

### Treatment Plan and Dose Modifications

The patients were treated using 3D-HDR-BT alone (BT group) or in combination with EBRT [combined-modality treatment (CMT) group]. The patients were classified as suitable for BT group provided that they met the following conditions: the tumor must have measured 10 mm or less, there must have been no metastases to the lymph glands. Of the 36 patients with rNPC, 17 were treated using the combined modality; briefly, patients received an equivalent dose of 50–66 Gy to the nasopharynx, with a fraction size of 2 Gy per day, metastatic neck nodes were 68 Gy (range, 64–70 Gy). After completion of EBRT, the patients were treated with a median dose of HRCTV for BT of 16 (range, 9–20) Gy at 2.5–5 Gy per fraction by using 3D-HDR-BT with a ^192^Ir source (microSelectron; Nucletron, the Netherlands). The remaining 19 patients in the BT group received a median dose of 32 (range, 20–36) Gy over 5–9 sessions by using 3D-HDR-BT alone with ^I92^Ir.

In the 3D-HDR-BT, intracavitary placement or implantation was selected according to the location and size of the residual tumor. The detailed methods have been described in our previous studies (11, 12). (1) 3D-CT-based interstitial brachytherapy was performed under the guidance of an electronic nasopharyngeal fibroscope. Two to four stationary ProGuide Sharp Needles (189.601 ProGuide Needle Set 6F, sharp) were inserted in the tumor tissues. The inserted needles were then fixed using buttons, CT imaging was performed using an interval of 2 mm, and the CT images were subsequently transferred to a treatment planning system (PLATO PBS 14.2). After delineation of the target volume, the images were transferred to PLATO Brachytherapy Planning. Finally, non-parallel needles were reconstructed and dose points were placed on the target surface. (2) For 3D-CT-based intracavitary brachytherapy, two to four customer-designed nasopharyngeal brachytherapy applicators were placed under local anesthesia with fiberoptic endoscopic guidance through the inferior meatus; the applicators were then immobilized using a thermoplastic mask. Other procedures were the same as described in Tang et al. (1).

### Follow Up

Patients were followed up for 4 weeks after BT treatment, every 3 months during the first 3 years and then every 6 months until death. Locoregional recurrence was defined as identification of recurrence within the primary site with or without regional involvement during the follow-up period. All events from the date of the end of BT to the date of recurrence or death were recorded. The late toxicities were graded according to the toxicity criteria of the Radiation Therapy Oncology Group (RTOG).

### Statistical Analysis

The date of final follow-up was March 14, 2018. The locoregional recurrence-free survival (LRFS), disease-free survival (DFS), and overall survival (OS) were calculated using the Kaplan–Meier method and were compared using the log–rank test. Data were analyzed using SPSS 20.0, and *p* < 0.05 was considered statistically significant.

## Results

### Patient Characteristics

Detailed characteristics of the patients (25 male, 11 female) are shown in Table 1. The median age at recurrence of NPC was 43.5 (range, 25–65) years. The median follow-up period for the entire group was 70 (range, 6–142) months. According to the 2002 cancer staging system of the American Joint Committee on Cancer, 32 patients were classified as T stage 1 (rT1), and 4 patients were classified as rT2. About 86.1% of the patients were rN0 disease. Histological examination showed that most of the patients (83.3%) had WHO type III disease. Of all patients, 17 were treated with EBRT+3D-HDR-BT (CMT group), while the other 19 were treated with 3D-HDR-BT alone (BT group).

**Table 1:** Baseline characteristics of patients.

Characteristics	**Patients (*n*)%**	
**AGE AT THE TIME OF RECURRENCE (y**)		
Median (range)	43.5 (25–65)	
**HISTOLOGY**		
WHO type I	3	8.3%
WHO type II	3	8.3%
WHO type III	30	83.3%
**GENDER**		
Male	25	69.4%
Female	11	30.6%
**rT-CLASSIFICATION**		
rT1	32	88.9%
rT2	4	11.1%
**rN-CLASSIFCATION**		
N0	31	86.1%
N1	3	8.3%
N2	2	5.6%
**ECOG**		
0	28	77.8%
1	7	19.4%
2	1	2.8%
**RECEIVED CHEMOTHERAPY**		
Yes	13	36.1%
No	23	63.9%

### Overall Survival

**Figure 1:**
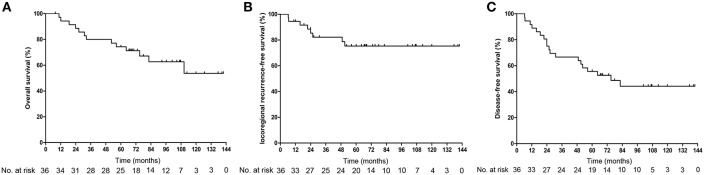
Kaplan-Meier estimated of the overall survival **(A)**, locoregional recurrence free survival **(B)**, and disease-free survival **(C)** of recurrent nasopharyngeal carcinoma patients.

**Figure 2:**
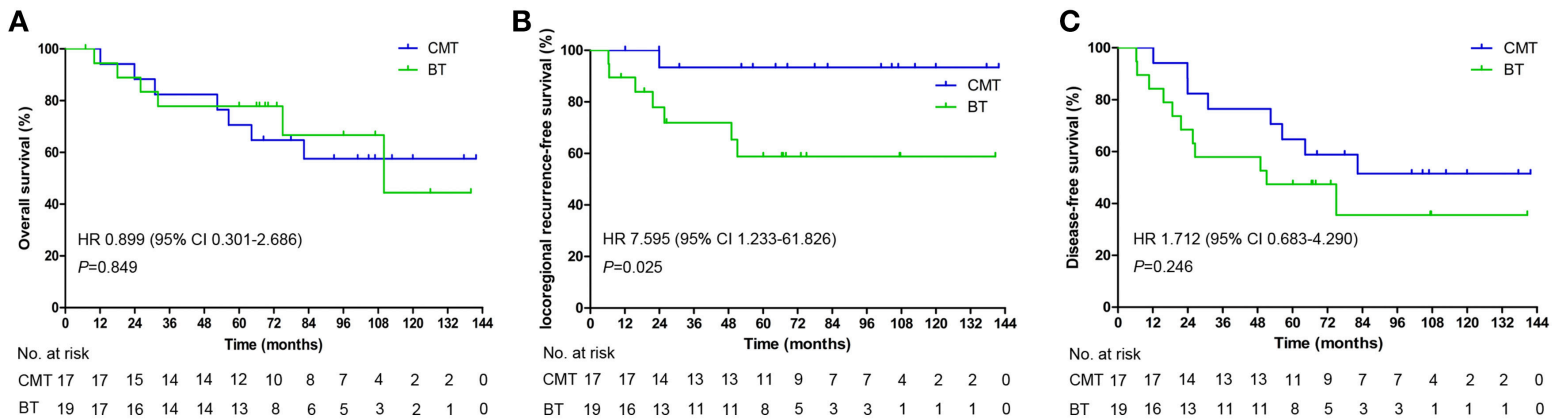
Kaplan-Meier estimated of the overall survival **(A)**, locoregional recurrence free survival **(B)**, and disease-free survival **(C)** in different groups.

In total, 13 (31.6%) patients (7, CMT group; 6, BT group) died during the follow-up period. Among the patients who were alive at the final follow-up, 17 patients had no evidence of disease and 6 patients experienced a relapse with or without active treatment. The mean OS was 102.3 months from the end date of BT [95% confidence interval (CI) 85.2–119.4], with estimated 3-, 5-, and 10-year OS rates of 80, 74.3, and 53.7%, respectively (Figure 1A). No significant differences were observed in OS between the CMT and BT groups [hazard ratio (HR), 0.899, 95%CI, 0.301–2.686, *p* = 0.849; Figure 2A]. The survival rates including CI are shown in Supplemental Table 1.

### Local Control

Tumor recurrence occurred in a total of 8 (22.2%) patients. Among them, 5 patients experienced local failure, 2 patients experienced regional failure, and 1 patient experienced both local and regional failures; 2 patients had distant metastasis (data not show). The mean overall LRFS was 111 months for the entire group. The 3-, 5-, and 10-year LRFS rates for the whole cohort were 82.2, 75.4, and 75.4%, respectively (Figure 1B). Among the 17 patients treated with the combined modality, 1 patient developed local recurrence after reirradiation, and subgroup analysis showed a 10-year LRFS of 93.8%. Of the remaining 19 patients who were treated with BT alone, 7 experienced local failure. The 10-year LRFS was 58.8%. Compared with patients who underwent BT alone, those who underwent CMT had a higher LRFS rate (Figure 2B, HR, 7.595, 95%CI, 1.233–61.826, *p* = 0.025).

### Disease-Free Survival

The median DFS was 75.03 months (95%CI 36.5–113.6), with 5-year and 10-year DFS of 55.6 and 44.2%, respectively (Figure 1C). The 10-year DFS for the CMT and BT groups was 51.5 and 35.5%, respectively (HR, 1.712, 95%CI, 0.683–4.290, *p* = 0.246; Figure 2C).

### Late Toxicities

We examined the incidence of late toxicity. Table 2 summarizes the number of patients with late toxicity and the grades for the entire cohort. The most common late toxicities were grade 1 headache, temporal lobe injury, hearing deficit, epistaxis, cranial neuropathy, xerostomia, trismus, and mucosal necrosis. Only 2 patients had grade 3 trismus, 1 patient had grade 3 temporal lobe injury, 1 patient had grade 3 hearing deficit and 1 patient had grade 3 xerostomia. No patients had grade 4 late toxicity.

**Table 2:** Late toxicities for patients with locally rNPC.

**Toxicities**	**Grade 0**	**Grade 1–2**	**Grade 3**
Headache	33	3	0
Temporal lobe injury	32	3	1
Hearing deficit	31	4	1
Epistaxis	32	4	0
Cranial neuropathy	34	2	0
Xerostomia	34	1	1
Trismus	31	3	2
Mucosal necrosis	33	3	0

## Discussion

The treatment of rNPC is difficult and challenging. Approximately 10–50% of patients with NPC experience recurrence after their primary treatment (13–15). A study by Lee et al. showed that the average recurrence interval in patients with NPC was 1.9 years (16). In addition, extremely poor prognosis has been observed in patients with local recurrence of NPC. The 5-year OS of patients with rNPC ranges from 12.6 to 37% (17, 18).

However, the development of efficient methods for the treatment of patients with rNPC continues to be a challenge. Several techniques have been used to treat rNPC, including external radiotherapy, stereotactic radiosurgery, nasopharyngectomy, and BT, alone or in combination with additional chemotherapy. The lesion of rNPC is often limited to the nasopharyngeal cavity, especially in patients at the rT1-2 stage and partial rT3 stage, and surgical treatment can be one of the salvage treatments. Although the 5-year survival rate for patients undergoing surgery ranged from 47 to 48.7% (19, 20), the incidence of associated complications was higher than 54% (21).

The treatment of rNPC requires a large dose of radiation due to the high radioresistance of tumor tissues. Although the optimal dose of radiation has not yet been established, Leung recommended a total dose of 60 Gy or more (22). However, a high radiation dose can induce severe early and late adverse reactions. Therefore, the optimal radiation dose and regimen in the treatment of rNPC are still debatable. Zheng et al. (23) reported the results of a study on 86 patients with locally rNPC who were treated with 3D-CRT. The 5-year actuarial local failure-free survival and OS were 71 and 40%, respectively, at a median dose of 68 Gy. Compared with conventional radiotherapy and 3D-CRT, IMRT more effectively covers the target area and minimizes damage to surrounding normal tissues. Han et al. (24) reported that reirradiation with IMRT in 239 patients with locally rNPC led to favorable local tumor control and prolonged patient survival; they reported a 5-year OS, LRFS, distant metastasis-free survival, and DFS of 44.9, 85.8, 80.6, and 45.4%, respectively.

Among the available therapies, BT has been widely used as salvage treatment of rNPC. Continual improvements in the afterloading machines have helped improve the treatment accuracy of BT. 3D-based image-guided BT enables the accurate delineation of the target organ and organs at risk. This approach has been widely used to treat patients with malignant tumors, such as prostate cancer (25), rectal cancer (26), breast cancer (27), and cervical cancer (28). BT can improve the local control rate in early stage NPC patients, thus it is effective in the treatment of the residual nasopharyngeal neoplasms after EBRT. In addition, compare with EBRT alone, BT has more favorable dosimetry, and delivers a decreased dose to the surrounding normal tissue, thereby leading to lower toxicity and improve local control.

Although there have been several reports addressing the use of BT in rNPC, they have been limited to studies based on retrospective data, limited patients number and short follow-up times. The results obtained have demonstrated that intracavitary HDR-BT can be a viable method for the treatment of patients with rNPC, especially those with early stage local recurrence (rT1 or rT2). For instance, Cheah et al. (29) reported that the 5-year LRFS, DFS, and OS of 33 patients with rNPC who were treated with ICBT alone or in combination with EBRT were 44.7, 38.8, and 28.1%, respectively. A study in which patients with rNPC were treated with ICBT using the HDR afterloading technique reported a median overall LRFS of 26 months, with 3- and 5-year LRFS of 50 and 25%, respectively (30). Additionally, Law et al. (31) reported that BT was an effective salvage treatment for patients with early T stage rNPC, and their 5-year local control and OS were 85 and 61.3%, respectively. Other reports of BT for locally rNPC are summarized in Table 3 (32–35).

**Table 3:** Other reports of brachythearpy for local recurrent nasopharyngeal carcinoma.

**Study**	**Treatment**	**Patients No**.	**Recurrence T-stage patient No**.	**Median Follow-up (months)**	**Outcomes (***%***)**			**Complications**
					**LC**	**OS**	**DSS**	
Shen et al. (32)	BT	30	rT1-2: 13 rT3-4:17	(2–38)	3y: 5.3	3y: 6.7	–	–
Yan et al. (33)	BT	39	rT1-2: 20 rT3-4:19	30 (5–68)	–	3y: 30.7	–	NP reaction (43.5%)
Kwong et al. (34)	BT	53	–	45	5y: 63	5y: 54	–	Palatal fistula (19%)
Syed et al. (35)	BT	41	–	82	5y: 59 10y:49	–	5y: 60 10y:60	Four patients died of RT complications

*LC, local control; OS, overall survival; DSS, disease specific survival; NP, nasopharyngeal; BT, brachytherapy*.

However, little data exists on the salvage treatment of rNPC with BT alone. To the best of our knowledge, the current study is the first to characterize the 10-year survival outcomes of patients with rNPC treated with salvage BT alone or in combination with EBRT. In this study, 17 patients underwent 3D-image-guided HDR-BT after EBRT, and 19 patients underwent 3D-image-guided HDR-BT alone. After completion of BT, the 10-year LRFS, DFS, and OS were 75.4, 44.2, and 53.7%, respectively. These results suggested that 3D-image-guided HDR-BT was effective for treating local rNPC. Approximately 22% of our study population experienced local failure, and two patients were diagnosed with distant metastases. No significant difference was found in the 10-year OS and DFS between the CMT and BT groups. However, compared with the patients in the BT group, patients in the CMT group had higher 10-year LRFS (*p* = 0.025). Our results showed that the CMT and BT groups had equivalent efficacy in terms of OS and DFS. BT and IMRT have shown comparable response rates, but new questions have arisen regarding which one is the best salvage treatment for early-stage rNPC and whether IMRT can deliver a dose high enough to eliminate the need for a boost. According to our results, we believe that BT is an excellent option in patients with limited-volume tumor. In patients with larger tumor volumes, the combination use of IMRT and BT appears promising.

Mucosal necrosis and massive hemorrhage of the nasopharynx are the most severe late complications of BT as well as the leading cause of death after re-irradiation in patients with rNPC. However, apart from 3 patient who had grade 1-2 mucosal necrosis, such severe late toxicities were not observed in this study. Noteworthy, none of the patients had radiation-induced osteonecrosis. Moreover, previous studies have revealed that compared with conventional EBRT alone, a combination of EBRT and BT for treating NPC was associated with significantly fewer complications (22, 36).

This study has several limitations. The main limitation is the retrospective nature of this study. In addition, this was a single-institution experience and small sample size.

In conclusion, 3D-HDR-BT achieves favorable clinical outcomes and acceptable late toxicities in treating patients with locally rNPC, and it should be considered an option in reirradiation alone or in combination with EBRT for patients with rNPC.

## Author Contributions

XC conceived, designed and supervised the study. YaL and YO collected and analyzed the data. ZL, YoL and KC provided technical assistance with the study. XC and YaL wrote the manuscript.

### Conflict of Interest Statement

The authors declare that the research was conducted in the absence of any commercial or financial relationships that could be construed as a potential conflict of interest.
