# Supplementary material for: Long-Term Outcomes of Three-Dimensional High-Dose-Rate Brachytherapy for Locally Recurrent Early T-Stage Nasopharyngeal Carcinoma
Source: Front Oncol. 2019 Apr 26;9:278. doi: 10.3389/fonc.2019.00278 (PMC6524703; doi:10.3389/fonc.2019.00278)
Supplement: Supplementary file 1 [file Table_1.doc]

Supplemental Table 1: Survival rate including confidence intervals for different treatment

|  | CMT | BT |
| --- | --- | --- |
| 5Y OS | 64.7%, 95%CI (0.420-0.847) | 77.8%, 95%CI (0.512-0.910) |
| 10Y OS | 57.5%, 95%CI (0.334- 0.816) | 44.4%, 95%CI (0.087-0.763) |
| 5Y DFS | 64.7%, 95%CI (0.420-0.874) | 47.4%, 95%CI (0.249-0.699) |
| 10Y DFS | 51.5%, 95%CI (0.270-0.760) | 35.5%, 95%CI (0.092-0.618) |
| 5Y LRFS | 93.3%,95%CI (0.617-0.990) | 58.8%,95%CI (0.351-0.825) |
| 10Y LRFS | 93.3%,95%CI (0.617-0.990) | 58.8%,95%CI (0.351-0.825) |

Abbreviation: Y=year; CMT= external beam radiotherapy+brachytherapy; BT=brachytherapy alone; LRFS=locoregional recurrence-free survival; DFS=disease-free survival; OS=overall survival; CI=confidence interval
